# Memristive Neural Networks for Predicting Seizure Activity

**DOI:** 10.17691/stm2023.15.4.03

**Published:** 2023-07-28

**Authors:** S.A. Gerasimova, A.V. Lebedeva, N.V. Gromov, A.E. Malkov, А.А. Fedulina, T.A. Levanova, A.N. Pisarchik

**Affiliations:** Researcher, Research Laboratory of Perspective Methods of Multidimensional Data Analysis, Institute of Information Technologies, Mathematics, and Mechanics; National Research Lobachevsky State University of Nizhny Novgorod, 23 Prospekt Gagarina, Nizhny Novgorod, 603022, Russia;; Associate Professor, Department of Neurotechnologies, Institute of Biology and Biomedicine; National Research Lobachevsky State University of Nizhny Novgorod, 23 Prospekt Gagarina, Nizhny Novgorod, 603022, Russia;; Laboratory Research Assistant, Research Laboratory of Perspective Methods of Multidimensional Data Analysis, Institute of Information Technologies, Mathematics, and Mechanics; National Research Lobachevsky State University of Nizhny Novgorod, 23 Prospekt Gagarina, Nizhny Novgorod, 603022, Russia;; Senior Researcher, Laboratory of Systemic Organization of Neurons; Institute of Theoretical and Experimental Biophysics of Russian Academy of Sciences, 3 Institutskaya St., Puschino, Moscow Region, 142290, Russia;; Junior Researcher, Laboratory of Brain Development Genetics, Research Institute of Neurosciences; National Research Lobachevsky State University of Nizhny Novgorod, 23 Prospekt Gagarina, Nizhny Novgorod, 603022, Russia;; Associate Professor, Department of System Dynamics and Control Theory, Institute of Information Technologies, Mathematics, and Mechanics; National Research Lobachevsky State University of Nizhny Novgorod, 23 Prospekt Gagarina, Nizhny Novgorod, 603022, Russia;; Head of the Laboratory of Computational Biology, Center for Biomedical Technology; Universidad Politécnica de Madrid, Madrid, 28223, Spain

**Keywords:** epilepsy, local field potentials, artificial neural networks, memristive devices

## Abstract

**The aim of the study** is to assess the possibilities of predicting epileptiform activity using the neuronal activity data recorded from the hippocampus and medial entorhinal cortex of mice with chronic epileptiform activity. To reach this goal, a deep artificial neural network (ANN) has been developed and its implementation based on memristive devices has been demonstrated.

**Materials and Methods:**

*The biological part of the investigation.* Young healthy outbred CD1 mice were used in our study. They were divided into two groups: control (n=6) and the group with induced chronic epileptiform activity (n=6). Local field potentials (LFP) were recorded from the hippocampus and medial entorhinal cortex of the mice of both groups to register neuronal activity. The LFP recordings were used for deep ANN training. Epileptiform activity in mice was modeled by intraperitoneal injection of pilocarpine (280 mg/kg). LFP were recorded in the awake mice a month after the induction of epileptiform activity.

*Mathematical part of the investigation.* A deep long short-term memory (LSTM) ANN capable of predicting biological signals of neuronal activity in mice has been developed. The ANN implementation is based on memristive devices, which are described by the equations of the redox processes running in the memristive thin metal–oxide–metal films, e.g., Au/ZrO_2_(Y)/TiN/Ti and Au/SiO_2_(Y)/TiN/Ti. In order to train the developed ANN to predict epileptiform activity, a supervised learning algorithm was used, which allowed us to adjust the network parameters and train LSTM on the described recordings of neuronal activity.

**Results:**

After training on the LFP recordings from the hippocampus and medial entorhinal cortex of the mice with chronic epileptiform activity, the proposed deep ANN has demonstrated high values of evaluation metric (root-mean-square error, RMSE) and successfully predicted epileptiform activity shortly before its occurrence (40 ms). The results of the numerical experiments have shown that the RMSE value of 0.019 was reached, which indicates the efficacy of proposed approach. The accuracy of epileptiform activity prediction 40 ms before its occurrence is a significant result and shows the potential of the developed neural network architecture.

**Conclusion:**

The proposed deep ANN can be used to predict pathological neuronal activity including epileptic seizure (focal) activity in mice before its actual occurrence. Besides, it can be applied for building a long-term prognosis of the disease course based on the LFP data. Thus, the proposed ANN based on memristive devices represents a novel approach to the prediction and analysis of pathological neuronal activity possessing a potential for improving the diagnosis and prognostication of epileptic seizures and other diseases associated with neuronal activity.

## Introduction

Epilepsy is characterized by spontaneous and unpredictable convulsions, which are often accompanied by worsening or loss of consciousness, psychological, vegetative, sensory, and motor symptoms [1]. The presently existing antiepileptic medications can satisfactorily control epileptic seizures in two thirds of patients [2]; in 8% of patients, epilepsy may be surgically eliminated. The remaining 25% of epileptic patients cannot be adequately cured with any presently available means.

Nowadays, medical treatment remains the most common method of epilepsy therapy. However, there exist a number of problems associated with insufficient efficacy and therapeutic safety of antiepileptic drugs. Some forms of epilepsy do not respond to medical therapy and are difficult to control. The Lennox– Gastaut syndrome, representing one of the forms of childhood-onset epilepsy manifesting itself during sleep, is referred to such forms [3-5]. Besides, a resistant form of epilepsy, which may develop due to the brain injury, infectious diseases, etc., is also refractory to the standard drug therapy [6]. It is worth mentioning that even at the proper level of the drug therapy efficacy, patients may periodically have some side effects: disorientation, depressive states, convulsions, slowing down effects, neurological deficit in the form of impairment of memory, attention, and concentration, problems with vision, hearing, and movement coordination [7-9]. In this connection, the search for the ways of prediction, correction, and treatment of epilepsy is one of the urging tasks facing the modern science requiring an interdisciplinary approach including neuroimaging technologies, gene investigations, current pharmacology, and mathematical methods such as machine learning of deep artificial neural networks (ANN).

In recent years, machine learning has proved to be a very effective tool for studying epilepsy. This can be explained by the fact that machine learning algorithms allow one to analyze a large amounts of data on brain activity [10-14] and medical images [15, 16], which, in its turn, helps better understand the nature of epileptic seizures, detect the regions of their origin and propagation, and develop the most effective plan of drug therapy taking into account individual patient’s characteristics [17, 18]. At the same time, it should be noted that the efficiency of deep ANN training depends directly on the quality of data used for training. Experimental data of neuronal activity in epilepsy may be acquired using various biological models. Here, the most preferable are the models using rodents (rats, mice), since rodents are capable of showing induced chronic epileptiform activity, which makes it possible to study pathological mechanisms of this disease.

In the current scientific literature, some interdisciplinary approaches to the investigation of neuronal activity in the biological models of epilepsy in rodents using machine learning are described in detail. For example, the results of classification of rodent neuronal activity in normal and pathological conditions have been presented in the papers [19, 20]. Of special interest are the investigations devoted to predictions of epileptic seizures based on the EEG data [21, 22]. The architectures of the deep ANNs vary from the convolutional neural networks [23] to transformers [24] and generative adversarial networks [25]. The authors [26] report a high accuracy obtained owing to machine learning in the task of predicting seizures in genetic models of absence epilepsy in rats based on recordings from corticothalamic regions [26].

It should be noted that deep neural networks have a great variety of parameters (weights) adjusted during training, which in its turn leads to high computational costs, which, with further development of this approach, may become excessive. In recent years, memristive architectures were widely used to solve this problem during implementation of various ANNs such as spiking neural networks [27, 28], multilayer neural networks [29-31], Hopfield neural networks [32, 33], convolutional neural networks [34, 35], and long short-term memory (LSTM) networks [36]. These new implementations of neural network architectures give an opportunity to obtain essential advantages from the standpoint of energy consumption, faster computation, and other important aspects. Memristive device can perform in-memory computations and a memristive crossbar array can accelerate vector-matrix multiplication. Therefore, the implementation of neural networks based on memristive devices is a promising way of solving the above problems.

Thus, owing to the advances in building ANNs, especially those using new energy-effective architectures (such as memristive crossbar arrays), new opportunities open up for effective prediction and analysis of pathological neuronal activity and, respectively, for designing novel state-of-the-art methods of predicting and treating epilepsy.

**The aim of the study** is to assess the possibility of predicting epileptiform activity using the data of neuronal activity recorded from the hippocampus and medial entorhinal brain cortex of mice with chronic epileptiform activity with the help of proposed deep artificial neural network, and to demonstrate the possibility of the implementation of this network using memristive devices.

## Materials and Methods

### Biological part of the investigation

The work complies with the Declaration of Helsinki (2013) and the Regulation of the European Parliament (86/609/EEC of November 24, 1986).

Young adult outbred male CD1 mice (n=12) with a 28–35-g body mass taken from the Clinic of Experimental Animals of the Institute of Theoretical and Experimental Biophysics of the Russian Academy of Sciences (Puschino, Russia) were used in the experiments. The mice were housed by two under the controlled conditions (22–24°C, 12-h light/dark cycle) with food and water *ad libitum*. The animals were randomly distributed into experimental (n=6) and control (n=6) groups. To induce *status epilepticus* in the model of chronic epilepsy, the awake mice were injected systemically with scopolamine (2 mg/kg intraperitoneally) and pilocarpine (280 mg/kg intraperitoneally) 30 min later.

The control mice of the same mass and age were injected a physiological solution in the same way. *Status epilepticus* was evaluated according to the Racine scale: stages 4, 5 (tonic-clonic seizures, round movements with posture loss and fall lasting not less than 1.5 h) were determined as the development of *status epilepticus*. Local field potentials (LFP) in the CA1 hippocampus field and in the medial entorhinal cortex, III (MEC III) layer, were recorded 1 month after the induction of *status epilepticus*. The procedure was done at the same time between 5:00 and 9:00 PM.

In the mice of the epileptic group, recording was performed in the interseizure period. Before the experiments, animals underwent surgical operation under general anesthesia (30 mg/kg of zoletil and 12 mg/kg of xylazine intramuscularly) in the Model 902 Small Animal Stereotaxic Instrument (David Kopf Instruments, USA). The body temperature was maintained with the help of electric pad, the cardiopulmonary condition during the operation was controlled by means of Oxy9Vet Plus pulse oximeter (Bionet, South Korea). Using the Brain Atlas (Paxinos & Watson, 1998), the depth electrodes (insulated Nichrome, 0.05 mm in diameter) were implanted into hippocampus (field CA1: AP (anteroposterior) was equal to –2.5 (rostro-caudal coordinate direction calculated from bregma); ML (mediolateral) was equal to 2 (mediolateral coordinate direction calculated from bregma); DV (dorsoventral) was equal to 1.5 (dorsoventral coordinate direction calculated from bregma)) and also into the medial entorhinal cortex (MEC III: AP=–3; ML=4.5; DV=5). A reference electrode was screwed into the occipital bone above the cerebellum. The entire complex was fixed on the head with acryl cementum. Within a week, the animals were recovering after the operation and getting used to the experimental environment. In this study, recordings of LFPs from hippocampus and MEC III were employed for ANN training, while the data on the study of the behavioral patterns in the process of neuronal activity registration were excluded.

### Mathematical part of the investigation

A deep neural network of the LSTM architecture capable of predicting biological signals of mice neuronal activity has been developed. Approaches have been shown permitting us to obtain circuit implementation of the network based on memristive devices, which can be described by the equations of redox processes in the memristive thin metal–oxide–metal films: Au/ZrO_2_(Y)/ TiN/Ti and Au/SiO_2_(Y)/TiN/Ti.

### Long short-term memory networks

**Figure 1.**
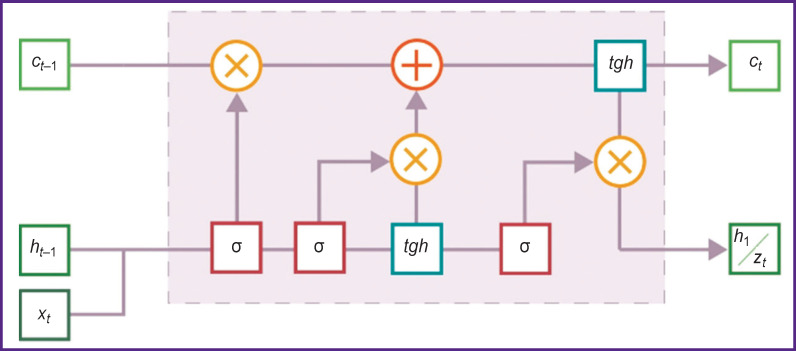
The architecture of the long short-term memory cell *z_t_* — output

A typical сell of the LSTM network is shown in Figure 1. It represents a recurrent network unit capable of memorizing values both for a short and long time intervals. The LSTM cell does not use the activation functions inside its recurrent components, therefore during ANN training using backpropagation through time, the stored value does not blur and the gradient does not vanish.

The cell operates in the following way. It has two hidden states: one represents a short-term memory *ht*, and the other — a long-term memory *ct*. Three filters regulate the information flow in and out of the cell. The cell also contains sigmoid blocks (σ) and blocks of hyperbolic tangents (*tgh*) called the gates.

The idea of a long-term memory consists in understanding by the total information from the short-term memory at the previous step *ht–*_1_ and from input *xt*, what information should be saved and what should not.

Let us consider first the information which we want to forget (not to keep further). The forget gate *ft* is responsible for this function. Its equation can be written as follows:


1 $$f_{t}=\text{σ}\left(W_{xf}x_{t}+W_{hf}{\text{h}}_{t\text{-1}}+b_{f}\right),$$

where σ is sigmoid activation function; *W_xf_*, *W_hf_* are the trainable weight matrices. Indices here and further have the following meaning. The first one indicates reference to a short-term memory *h* or input *x*. The second index denotes the reference to the gates. Thus, *W_xf_* is the trainable weight matrix corresponding to input *x* and forget gate *f_t_*. The trained biases are denoted as *b*, here and further the indices indicate reference to the gate. The *b_f_* is therefore the trainable bias corresponding to the forget gate *f_t_*. If component-wise multiplication (×) by the long-term memory state *c_t_*_–1_ gives 0, this information will be forgotten, if the result is 1, it will be saved.

The gate with sigmoid σ is used for the information we want to remember in order to understand, in which components of the long-term memory state *c_t_* useful information should be inserted:


2 $$i_{t}=\text{σ}\left(W_{xi}x_{t}+W_{hi}{\text{h}}_{t\text{-1}}+b_{i}\right).$$

The gate *g_t_* with hyperbolic tangent (*tgh*) is employed to select which information is to be saved:


3 $$g_{t}=tgh(W_{xg}x_{t}+W_{hg}{\text{h}}_{t\text{-1}}+b_{g}).$$

In other words, in the scheme of Figure 1, multiplication means selection of information, while addition is adding new information. Then, the final formula for changing the long-term memory *c_t_* will be as follows:


4 $$c_{t}=f_{t}c_{t-1}+i_{t}g_{t}.$$

To obtain the output *h_t_*, the gate of the output *o_t_* and the information from the long-term memory *c_t_* are used:


5 $$h_{t}=o_{t}\times tgh(c_{t}),$$

where *o_t_*=σ(*W_xo_x_t_*+*W_ho_h_t_*+*b_o_*).

Deep ANNs containing LSTM cells are called long short-term memory networks (LSTM networks). The deep neural LSTM network used in this study has the following architecture. The first input layer is a linear layer translating the input information into the 100-dimensinal feature space. Then come two layers of LSTM cells. The result is projected by the linear output layer. The weight matrices *W* and shifts *b* are trained using the method of error backpropagation using mean squared error (MSE) loss function:


$$\text{MSE}=\frac{1}{N}\sum_{n=1}^{N}{\left(y_{n}-{\hat{y}}_{n}\right)}^{2},$$

where *N* is the number of samples, *y_n_* — true amplitude value for the n^th^ sample, and *ŷn* is the predicted value for the n^th^ sample.

The LSTM networks are suitable for classification, processing, and prediction based on the time series data since there may be intervals of unknown duration between important events in the time series. Relative insensitivity to the window length is also an advantage of the LSTM networks over the common recurrent networks, hidden Markov models, and other methods of machine learning in tasks with sequences in many applications. In our previous paper [37] we have tested the ensemble consisting of the neural networks of diverse types (the feed forward networks, reservoir computing, and LSTM networks) to predict extreme events and chaotic dynamics using the time series data.

### Memristive devices

As a hardware, a weight matrix of the LSTM cell may be implemented using arrays of memristor crossbars [35, 36]. Separate memristive devices of the metal-oxide-metal type [38] represent thin-film structures, whose conductivity alters by several orders of magnitude when voltage is applied. A memristive device is a resistor with a memory, which is able to retain the received state: low or high ohmic, which points to the so-called resistive memory. To model the behavior of the laboratory memristors, we used a standard approach describing the reduction-oxidation processes running when electrical voltage *u* is applied. The memristor state *w* changes due to oxygen ions migration at the increase of the effective migration barrier *E_m_*. Migration in its turn is provided by Joule heating *kT* and applied electric voltage *u*. The total current density via the memristor represents the sum of linear *j_lin_* and nonlinear *j_nonlin_* constituents. The first one corresponds to the ohmic conductivity with resistivity ρ, the second one is determined by the transport of the charge carrier through the defects in the insulator region not occupied by the filaments including the region of the filament rupture. The current is carried according to the Poole–Frenkel mechanism with an effective barrier *E_b_*.

In the present study, we used equations for memristive switching (6), which were derived in paper [39].


6 $$\{\begin{array}{l} j=wj_{lin}+(1-w)j_{nonlin}\\ j_{lin}=u/\rho \\ j_{nonlin}=u\exp(B\sqrt{u}-E_{b})\\ \frac{dw}{dt}=\{\begin{array}{l} A\exp(-E_{m}-\alpha u)(1-{\left(2w-1\right)}^{2p}),u<u_{set}\\ 0,V_{set}<u<V_{reset}\\ -A\exp(-E_{m}-\alpha u)(1-{\left(2w-1\right)}^{2p})u<u_{reset} \end{array} \end{array}$$

Parameters *A*, *B*, *α* are taken from the experimental data. Parameters *u_set_* and *u_reset_* are threshold voltages of the memristive structure switching. Parameters *E_b_* and *E_m_* represent effective internal parameters characterizing different films (Au/ZrO2(Y)/TiN/Ti, Au/SiO2(Y)/TiN/Ti), *p* is a positive integer, which provides a zero value *w* beyond the interval (0, 1).

Implementation of memristors in the crossbar arrays for vector-matrix multiplication gives a high accuracy of computing at a small size of the device itself.

### Memristive neural networks

**Figure 2.**
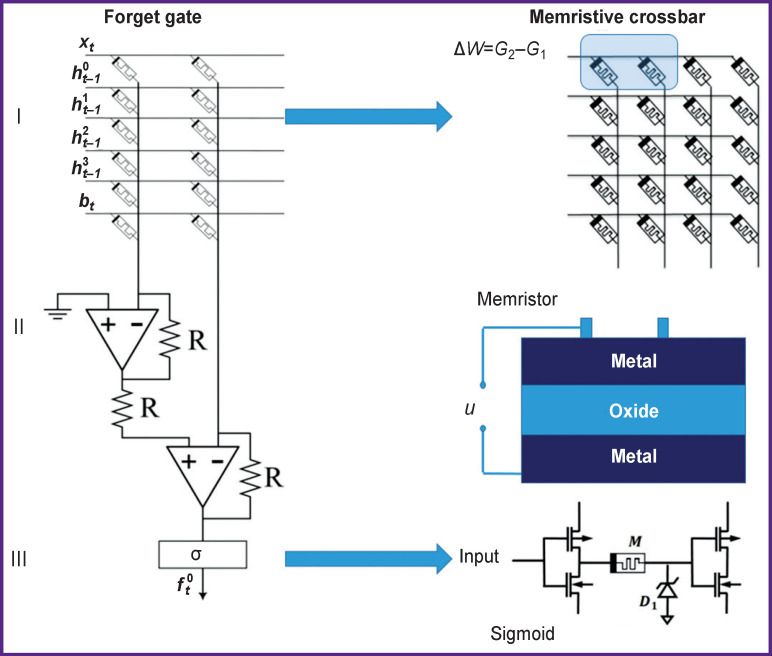
Schematic diagram of vector-matrix multiplication for the forget gate: I — memristive crossbar, where *W* is a weight matrix, *G* — conductivity of the memristive device; II — memristor structure, where *u* is applied voltage; III — the implementation scheme for the sigmoid function

As a weight in the LSTM cell may take positive or negative values, it may be presented as a conductivity difference of two memristors Δ*W*=*G*_2_–*G*_1_ [40]. This doubles the number of memristors in the matrix. The implementation of the LSTM cell forget gate is shown in Figure 2. The similar approach may be used for building the rest of the gates.

For subsequent hardware implementation of the memristive neural network, elements such as memrisive device, memristive crossbar, sigmoid, and hyperbolic tangent were realized in the Simulink program taking into consideration the parameters of the laboratory memristors. The schematic diagram of the forget gate (see equations (1)–(5)) is presented in Figure 2. Sigmoid and hyperbolic tangent were implemented on transistors [41]. In this case, the property of differential amplifier was used: a gradual and smooth increase in the output voltage when the differential input is in the desired range.

## Results

The designed deep neural network was trained on the data of neuronal activity of mice with epilepsy obtained in laboratory conditions. Three types of numerical experiments have been carried out. The data were preprocessed using Gaussian filtering in all cases to eliminate the noise. In the first experiment, we used the data of the long LFP recording for one mouse with epileptiform activity, which were divided into the training and testing samples in the ratio 4:1. Then, the data were normalized so that their mean value was equal to zero, and the variance to 1. Next, the data were converted to the “time sequence–response” format. For example, 20 time counts were supplied to the model input, and the 21^st^ was used as a response. Our LSTM network was trained on these sequences. For single-step prediction, the testing part was preprocessed in the described way.

For multistep prediction, the model response to the previous step was iteratively added to the sequence to the network input.

In the experiments of the second type, LFPs of all mice were used as the data. Each recording was divided into the training and testing sample in the ratio 4:1, then followed the same sequence of actions as in the first experiment.

In the third experiment, LFP recordings were used as training data for all mice except one, which served as a test mouse.

The quality of epileptoform activity prediction was evaluated using the root-mean-square error (RMSE) metrics:


$$\text{RMSE}=\frac{1}{N}\overset{N}{\underset{n=1}{\sum }}{\left(y_{n}-{\hat{y}}_{n}\right)}^{2},$$

where *N* is the number of samples, *y_n_* — true amplitude value for the n^th^ sample, *ŷ_n_* — predicted amplitude for the n^th^ sample.

**Figure 3.**
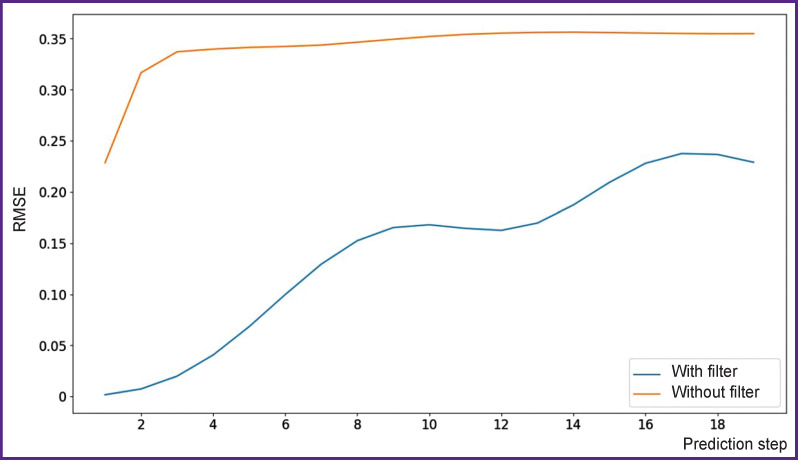
The value of RMSE metrics depending on the prediction step The orange curve corresponds to numerical experiments where data were used with no filtering, blue curve — to the experiments with the data after Gaussian filtering

**Figure 4.**
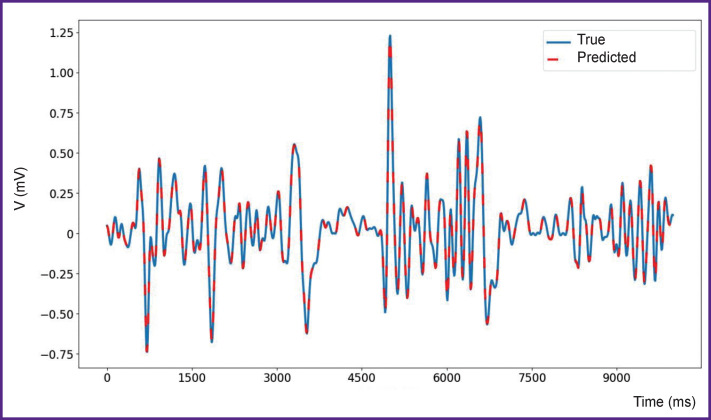
True (*blue line*) and predicted (*red line*) values for one step of prediction of local field potentials for a mouse with epileptiform activity

**Figure 5.**
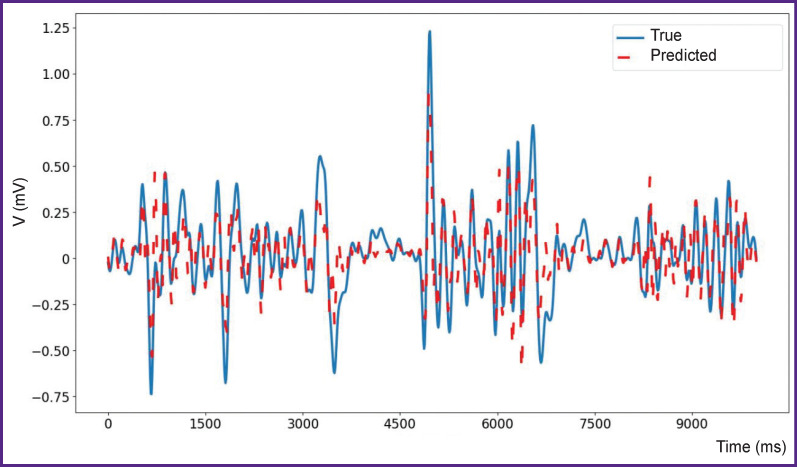
True (*blue line*) and predicted (*red line*) values for five steps of prediction of local field potentials for a mouse with epileptiform activity

The results of numerical experiments are presented in Figures 3–5. As seen from Figure 3, the quality of time series prediction strongly depends on the presence of a data filter and the prediction step. Single-step prediction is sufficiently accurate (RMSE=0.019), although some errors of incorrect prediction of the event amplitude are observed. The prediction accuracy decreases significantly with the increase of the prediction step size. True and predicted values for a single-step prediction of the time series with epileptiform activity are shown in Figure 4. Similarly, the true and predicted values for five-step prediction of the time series with epileptiform activity are presented in Figure 5. It is also seen in the Figures 3–5 that with the increase in the number of steps, the quality of prediction of high-amplitude values of the time series drops in the first place beginning with the fifth step. It should be noted that accurate prediction of high amplitude events is especially important for the prediction of seizure activity. Here, the events with the amplitude exceeding the mean value by more than 5 standard deviations are considered high-amplitude events [38]. Notable, that for the prediction by less than 5 steps ahead, precision equal to 100 and recall to 76 may be obtained for high-amplitude events. These results agree with the previous data [42] on predicting epileptiform activity. Thus, the proposed network is able to predict precisely enough the appearance of epileptiform activity 40 ms before its onset.

## Discussion

Early prediction of epileptic seizures is very important for preservation of patient’s health and life. Several seconds are enough for the individual to take a comfortable position and not to fall suddenly injuring himself, or not to create emergency situation, e.g., when driving a car.

Although in recent years, a significant progress was achieved in the detection of specific patterns in time series of neuronal brain activity [41-45], investigations in predicting seizure (focal) epileptiform activity were not fruitful. Nevertheless, several successful attempts were made in this field using various approaches. For example, Li et al. [43] have applied a permutation entropy method for prediction of seizure activity in rats. The authors succeeded in obtaining the mean prediction time of 4.9 s. Another approach based on the statistical properties of brain activity and the theory of extreme events [44] allowed for prediction of convulsion occurrence in WAG/Rij rats 7 s before their beginning [45]. A highly precise prediction of epileptic seizures prior to their onset by analyzing the LFP data using methods of deep neural network will provide the opportunity to expand essentially the possibilities of therapy and create the diagnostic methods enabling to detect early disorders in the patient’s rhythmic brain activity.

Usage of memristive elements as a hardware platform for ANN implementation is able not only to solve a number of technical problems typical for ANN (great memory and energy consumption for training) but will help develop portable therapeutic devices for tracing patient’s brain activity, and in case of threatening conditions, apply optimal external stimulus to eliminate this state. Creation of such devices in combination with traditional therapy will provide the opportunity to improve the quality of patient’s life, reduce morbidity and mortality rate.

## Conclusion

In the present study, we have used a deep artificial neural network to predict pathological neuronal activity, in particular, chronic epileptiform neuronal activity in mice. The main advantage of this approach consists in application of memristive devices as a hardware platform for implementation of the artificial neural network. This approach provides fast and energy-efficient computing.

The designed artificial neural network demonstrates the ability to predict seizure (focal) epileptiform activity prior to its actual occurrence. This study is of great importance for early epilepsy diagnosis and treatment. The results obtained contribute to a deeper understanding of the mechanisms of epileptic seizure development in general.

Usage of deep artificial neural networks and memristive devices open up new prospects for the development of novel and more precise methods of predicting epileptiform activity and other neurological diseases. Application of a more detailed mathematical analysis may help improve the accuracy and reliability of predictions.

However, application of the obtained results in clinical practice requires additional investigations in humans. Such studies will allow the specialists to confirm and summarize the results and evaluate the suitability of the developed methodology for predicting and diagnosing epilepsy in patients.
